# Same place, different time: Leopards (*Panthera pardus kotiya*) employ temporal partitioning as a co-existence mechanism in a human-dominated, unprotected agricultural landscape in Sri Lanka

**DOI:** 10.1371/journal.pone.0335296

**Published:** 2026-09-09

**Authors:** Andrew M. Kittle, Anjali C. Watson

**Affiliations:** The Wilderness & Wildlife Conservation Trust, Colombo, Sri Lanka; University Centre Sparsholt, UNITED KINGDOM OF GREAT BRITAIN AND NORTHERN IRELAND

## Abstract

As humans and wildlife increasingly overlap on a rapidly changing planet, it is important to understand co-existence mechanisms in unprotected landscapes. This is especially so for large carnivores, with which humans share an uneasy history. In Sri Lanka’s tea estate-dominated Central Highlands, a dense, widespread human population shares the landscape with the island’s apex predator, the Sri Lankan leopard (*Panthera pardus kotiya*). This study used remote cameras to determine leopard density, generalized linear models to investigate leopard space-use in relation to key anthropogenic, ecological and environmental variables, and fitted kernel density curves to identify temporal activity patterns in this unprotected, human-dominated region. Adult leopard density (8.82/100 km^2^) was lower here than in key Sri Lankan National Parks, but not significantly, suggesting good leopard habitat suitability. Spatially, leopards did not avoid humans but incorporated anthropogenic factors into space-use decisions, with areas of intermediate human and dog presence disproportionately used. Male leopards were more likely to occur at intermediate distances from human settlements, perhaps balancing risk with efficiency while navigating this shared landscape. Female leopards biased space-use towards areas of high natural prey availability, aligning with their need to provision cubs. Temporally, leopards were significantly less diurnal and more active at dusk than their protected counterparts. This was likely due to temporal partitioning from diurnally active humans, an adaptation which allows for human-leopard co-existence. This is the first comprehensive study of leopards outside protected areas in Sri Lanka, and highlights the value of unprotected landscapes for long-term leopard conservation. To foster human-leopard co-existence it is necessary to maintain current human activity patterns which allow for temporal partitioning; ensure existing natural prey resources remain to prevent leopards switching to domestic prey species; and reduce/eliminate the use of wire snares which transcend the effect of temporal partitioning by de-coupling persecution risk from human presence.

## Introduction

Protected areas (PAs) represent vital refuges for wildlife and are key components of long term global conservation efforts [1–3]. However, the vast majority (~85%) of the world’s terrestrial area is unprotected [4], with wildlife relying on these unprotected landscapes for their survival [5–7], either for migration [8], dispersal [9], foraging [10], or as part of their daily range [11]. Unprotected landscapes pose greater risks to wildlife than PAs, mostly due to their widespread use by humans and the land use changes they affect [12]. Relatively little is understood, however, about how wildlife utilize these typically human-dominated landscapes, especially in relation to what is known about their behavior and ecology within PAs. With the world’s human population and ecological footprint continuing to grow [13], and climate change impacts expected to increasingly see wildlife species move out of their traditional regions [14], it is imperative to better understand how shared spaces are used.

Apex predators have a long and complex relationship with humans [15], and are often viewed negatively [16,17] making them frequent targets of persecution [18}. Therefore, understanding the mechanisms by which co-existence with humans can be promulgated is key to their long-term viability. These carnivores can play vital roles within ecosystems and their disappearance can have cascading effects across trophic levels, yet they are being extirpated at an alarming rate [18]. Just as the study of human-wildlife conflict has shed light onto important factors that create tension between people and the animals with which they share space [19], equal consideration must be given to understanding the factors that promote human-wildlife co-existence so that these factors can be incorporated into wildlife policy, planning and management [20].

Unprotected areas typically have diminished natural prey availability [11], a key determinant of large predator home range size and density [21]. These areas also tend to be associated with increased risk [22] and often have regions – e.g., city centres, agricultural expanses – incompatible with large carnivore use, requiring individuals to use larger areas to accomplish basic activities such as foraging, mating and rearing young. It might therefore be expected that carnivore densities outside PAs are lower than those within comparable, well-protected PAs. A key initial consideration for the management of wildlife in unprotected areas is to understand how many reside there.

Spatial partitioning between humans and wildlife has long been key to successful, broad-scale co-existence, with the concept of wildlife spaces distinct from human spaces underlying the establishment and maintenance of Protected Areas [23]. However, as wild animals cross artificial boundaries and human populations expand, in much of the world humans and wildlife share space [24]. Even outside PAs, apex predators often employ fine scale spatial avoidance of humans and/or human infrastructure, such as the avoidance by lynx (*Lynx lynx*) of dense road networks within their home ranges in Norway [25], the avoidance of human settlements by tigers (*Panthera tigris*) in China [26] or the avoidance of areas of high human presence by lions (*Panthera leo*) [27]. In some landscapes, however, spatial partitioning is impractical due to high human densities or a widespread human footprint across space [13], and here it is increasingly seen that top predators employ temporal partitioning to avoid people [28,29].

Sri Lanka is a relatively small (65,610 km^2^), densely populated (336/km^2^), mostly rural (~80%) island that – together with India’s Western Ghats – is one of the world’s 36 global biodiversity hotspots [30]. In Sri Lanka, the leopard (*Panthera pardus kotiya*) is a threatened, endemic sub-species and the island’s apex predator [31]. Despite a low estimated population of < 1000 mature individuals [32], leopards in Sri Lanka remain extant in all climatic zones and are present in both protected and unprotected areas [31].

In Sri Lanka’s Central Highlands – a United Nations Educational, Scientific, and Cultural Organization (UNESCO) World Heritage site based on its high levels of biodiversity and species endemism [33] – leopards reside in and around the unprotected, tea estate-dominated landscape [34; S1 Fig] where every year several animals are killed due to human persecution [35]. Prior to the widespread clearance of forest for the cultivation of first coffee (1830–1880) and then tea (*Camillia sinensis*; 1880 – present) during British colonial times, the Central Highlands were blanketed in thick sub-montane and montane forest [36], of which mostly small, isolated patches remain. Despite anecdotal tales of leopards in and around these forest patches from colonial days to the present, and despite research on the leopard being conducted in the region’s most iconic PA, Horton Plains NP [37,38], almost nothing is known about the behavior and ecology of leopards in these unprotected tea estate landscapes (but see [34]).

The objective of this study was therefore to improve understanding both of leopard ecology and behavior outside protected areas in Sri Lanka and of how these apex predators have adapted to sharing space with humans. Specifically, this study tested three hypotheses related to human-wildlife co-existence: 1) Human-dominated landscapes are sub-optimal for leopards. If this is supported, leopards should be observed living at a lower density in this landscape compared to within the island’s foremost protected areas. 2) Leopards employ spatial partitioning to reduce threats from humans. If this is supported, leopards should be avoiding areas of high human use and/or infrastructure. 3) Leopards employ temporal partitioning to reduce threats from humans. If this is supported, less diurnal leopard activity should be observed in this unprotected, heavily cultivated landscape compared to protected ones on the island.

## Materials and methods

### Study area

**Fig 1:**
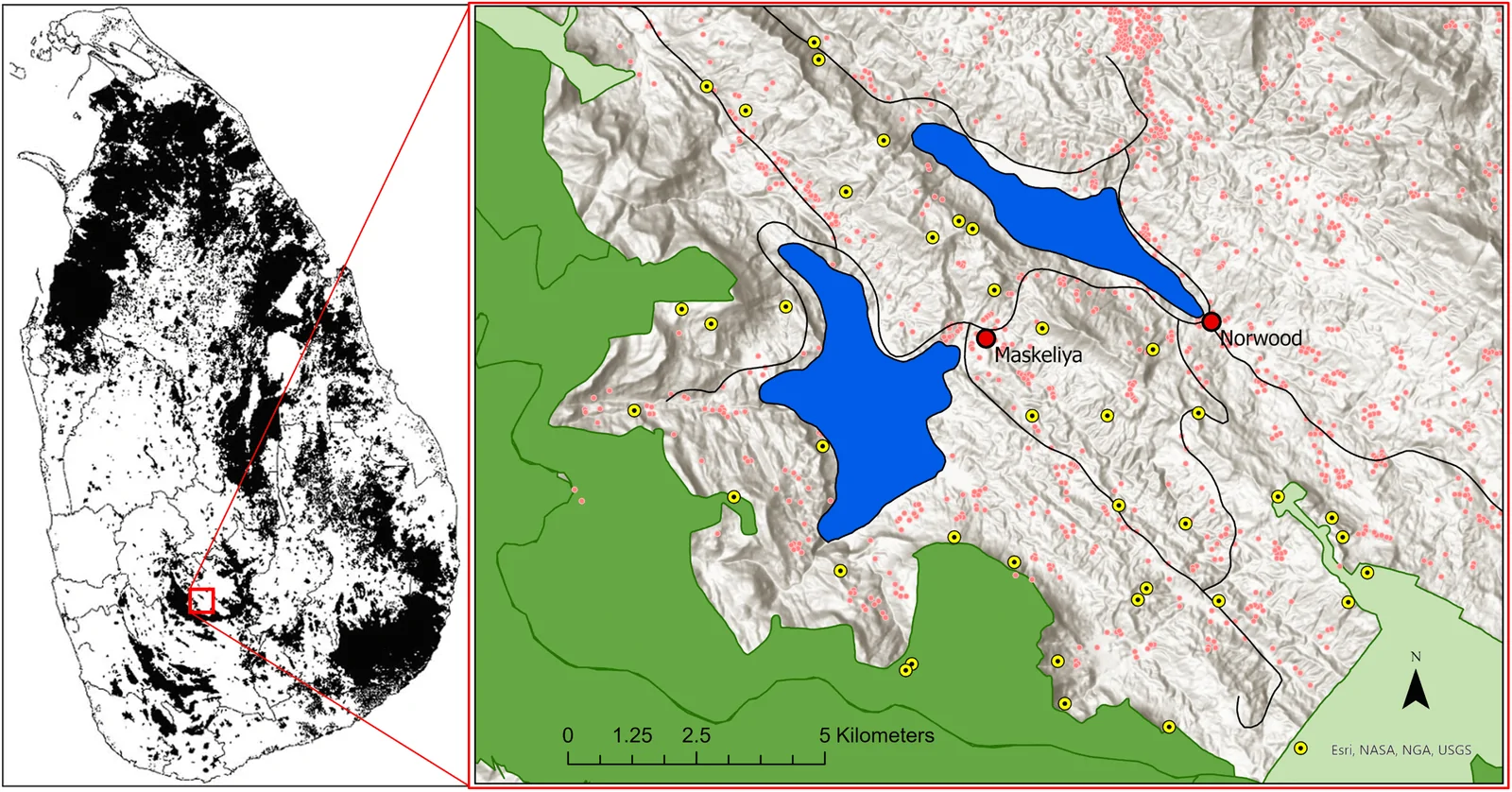
Map of Sri Lanka showing current forest cover (black shading) with the inset highlighting the study area. The yellow dots with black centres are remote camera locations (N = 40) utilized during this study. The two large hydro-electric reservoirs can be seen in blue, with the larger Moussekelle reservoir surrounded by remote cameras and the narrower Castlereagh reservoir on the north-eastern edge of the camera array. The dark green along the southern and western regions is the Peak Wilderness Protected Area complex with the lighter green being proposed but currently undeclared forest reserves. The two main towns within the study area – Maskeliya and Norwood – are depicted by labelled dark red circles with other areas of human infrastructure in the form of buildings (light pink dots) and roads (black lines) also shown. The map was created in ArcGIS Pro version 3.4 using the base map Esri’s World Hillshade layer [41].

This study was conducted in the southern region of the Central Highlands of Sri Lanka, in the Upper Kelani Valley catchment area surrounding the Moussekelle reservoir, immediately north of the Peak Wilderness Sanctuary (Fig 1). The region is characterized by vast expanses of tea cultivation, interspersed with fragmented, often secondary forest patches, dense shrub patches where tea has been “released”, and Eucalyptus (*Eucalyptus sp.)* and Pine (*Pinus caribaea*) plantations [39]. The region is within Sri Lanka’s sub-montane wet zone with annual rainfall > 2500 mm, which falls throughout the year but peaks in October-November (> 300 mm/month) and April and May (250–300 mm/month), roughly coinciding with annual monsoons. The dry season (< 150 mm/month) runs from January through March. Average temperatures range from 18° - 30°C, but can dip to 10°C on higher slopes at night [40]. Elevation in the study area ranges from 1000–1800 MASL, with steep ridgelines running between shallow, flattened valleys where rivers and streams flow.

In this region of the Central Highlands the tea industry is dominated by large Regional Plantation Companies (RPCs). RPCs typically run several neighbouring estates, divided themselves into several (~2–8) Divisions, each including at least one community comprised of estate workers and their families. Larger towns are also widespread, typically along valley bottoms where waterways and main roads are also located. As such, the human footprint in this landscape is large and extensive, making broad-scale spatial separation between humans and wildlife impossible. The human population density of the Nuwara Eliya District in which the study area is located is > 400/km^2^ [42].

### Leopard density

To estimate leopard density, we set 40 remote camera stations throughout the Central Highlands landscape surrounding the Moussekelle reservoir from mid-August – end-November 2016 (Fig 1; S1 Table in S1 Appendix). Fieldwork was conducted under the Department of Wildlife Conservation (DWC) permit number WL/3/2/1/4/1. Since adult leopards across continents and landscapes frequent roads and other linear pathways while patrolling their territories [37,43–46], cameras were set adjacent to and facing unpaved tea estate roads, walking paths or animal trails. Twenty of the stations comprised two cameras, one either side of these trails, with the balance 20 stations having a single remote camera. There was some variation in the distance from trails (1 – 3m) of cameras and camera height (30 cm – 1 m) due to the heavily cultivated landscape which necessitated use of infrequent trees or sturdy tea bushes, but cameras were nevertheless set to ensure photo-capture of leopards and their main prey [47].

The study was divided into 3 rounds, with round 1 employing 12 camera locations across 36 days, round 2 using 14 camera locations across 35 days and round 3, 14 camera locations across 44 days (Table A in S1 Appendix). Total study duration was therefore 115 days, which is within the time period estimated to ensure a closed population for big cats [47,48]. Furthermore, the closure test in the ‘secr’ package was employed to verify the assumption of closure [49]. The un-buffered camera array covered ~ 140 km^2^ (Fig 1) with locations spaced an average of 1.2 km apart, to ensure all prospective leopards were exposed to detection in this unknown landscape and optimize the trade-off between area coverage and photographic recaptures while ensuring a closed population [47]. To ensure sufficient image quality for accurate identification of individuals, we used Scoutguard SG565F (Boly Inc., Shenzen, China) incandescent flash cameras with sensitivity set to “normal” and minimal camera delay (“0”) to ensure capture of multiple animals moving together.

Photo-captured leopards were individually identified using their unique spot patterns, with age and sex categorized based on morphological differences [46]. For each individual a capture history was developed but only mature leopards (estimated to be ≥ 2 years old) were used for population density analysis. Both flanks of mature leopards were almost all clearly photo-captured, except one adult female whose left flank only was photographed and three unsexed individuals whose right flanks only were photographed. Since the three unsexed individuals were not clear images, we elected to utilize only individuals who had been photographed on the left flank. Employing only a single flank has the potential to result in individuals being excluded which can impact density estimates. However, the fact that the three images excluded were lacking in clarify sufficient to unambiguously identify or sex the individuals, alleviates this concern.

We used spatially explicit capture-recapture (SECR) models within a maximum likelihood (ML) framework (*secr* 4.6.0 in R) [50] to estimate leopard density. The SECR process, which fits spatial models of the population and detection process to individual spatial detection histories, is considered unbiased by edge effects or incomplete detection [51]. SECR models were constructed using count detectors to account for multiple or repeat individuals captured by each trap on each occasion. Given the variation in the number of days that individual camera stations were active, we incorporated varying effort by valuing complete 24-hour camera occasions as 2, 12-hour occasions as 1, and inactive days as 0 [46,52]. We fitted a half-normal detection function with Poisson distribution, which is the most commonly employed function in spatial capture-recapture analysis [53]. This function describes the probability of capture (*P*) of individual *i* at trap *j* as a function of the distance (*d*) from that individual’s activity centre to the trap using the equation *Pij* = *g0* exp (-*dij*^2^/2σ ^2^)), with *g0* the probability of capture at home range centre and *σ* a spatial parameter related to home range size [53]. Since our study area is centred around two large hydro-electric reservoirs (Fig 1), we created a habitat mask with a 15 km buffer around the remote camera array and with the reservoirs defined as “non-habitat” and removed. We did not consider any other areas “non-habitat” as leopards in this heavily fragmented landscapes are known to occasionally be seen even in human communities. We used *secr*’s “suggest.buffer”, “make.mask” and “check.mask” functions to determine the buffer size that would be utilized for further analysis (Table B in S1 Appendix) [51,54].

Variation between sexes in both range size and use structures leopard spatial dynamics [55] and male leopards in Sri Lanka typically utilize much larger areas than females [46,48,56]. Therefore, we fit 2-class hybrid mixture models (hcov in *secr*) with sex modelled as a covariate [48,54,57] allowing for varying capture parameters (*g0* and *σ*) between sexes. Using Akaike’s Information Criterion for small sample sizes (AIC*c*) as well as Akaike weights (AIC*cwt*) [58] to compare them, we tested 4 models: 1) a null model without variation in capture parameters; 2) a model allowing sex-specific variation in *g0*; 3) a model allowing sex-specific variation in *σ*, and 4) a model allowing sex-specific variation in both capture parameters.

### Leopard site utilization

At each of the 40 camera stations, as the dependent variable, we determined the total number of leopard observations, inclusive of all age/sex classes, which were considered to be independent if >1 hour passed between detections of the same individual at the same site. If leopard detections <1 hour apart were of different individuals– based on age, sex and/or individual identification – these were also utilized for analysis.

To investigate the factors driving occurrence patterns, each location was characterized by ecological and anthropogenic attributes selected to represent one of four specific underlying factors: human-leopard co-existence, habitat suitability, landscape topography and prey availability. The human-leopard co-existence variables were 1) the relative abundance index of humans at each camera location (RAI = (# of human or vehicle detections/# remote camera 24-hour surveillance periods) * 100), 2) the RAI of domestic dogs, 3) the distance (m) to nearest human settlement, and 4) the distance (m) to nearest road. Distance to human settlement was determined using the “distance measure” tool on Google Earth because many estate communities are small clusters of dwellings which are not represented on available GIS layers, but are visible on Google Earth maps. Distance to road employed GIS shape files acquired from the Sri Lanka Survey Department [59] of paved primary (Main road A Grade) and secondary (Minor road B Grade) roads with the distance determined using the “Near” tool under “Proximity” analysis in ArcGIS. Habitat suitability metrics were based on the importance of protected areas (PAs) and forest cover for leopard distribution in Sri Lanka [31]. Distance to PA was determined in the same way as distance to roads above, only utilizing the Sri Lanka Protected Area layer [57]. Distance to forest was determined in the same way as distance to settlement above, since again, the grain of available GIS layers was not sufficiently fine to allow identification of small forest patches in this landscape. For this analysis, forests were considered all treed landscapes inclusive of plantation forests*,* which here are typically Eucalyptus-dominated stands originally planted to provide fuelwood for the tea factories. Natural and plantation stands were treated as the same since leopards are adept at utilizing a wide range of habitats [60] including plantation patches, which are especially important in landscape mosaics [61] despite their inferior quality from a biodiversity perspective. Landscape topography was represented by a measure of elevation (MASL) taken with a hand-held GPS unit at each remote camera location. Prey availability was represented by black-naped hare (*Lepus nigricollis*) RAI and red muntjak (*Muntiacus muntjak*) RAI as both species are widespread in the tea estate landscape and important prey species here [62], with the former the most available on the landscape [62] and the latter the only potential prey species whose weight is within the range “highly preferred” by leopards globally (23–25 kg) [63]. The original variable list contained two metrics of available prey biomass – total biomass and biomass of species within the weight range (10–40 kg) of leopard preference [63] – however correlation analysis revealed these variables to be highly correlated (r > |0.8|) with each other and several other independent variables, so were dropped from analysis. The final list of independent variables all had correlation coefficients < |0.7| (Table C in S1 Appendix). To test for non-linear relationships, whereby leopards may be more likely to be found at intermediate levels of a given variable [60,64], we employed the quadratic of four independent variables: Human_RAI, Dog_RAI, Distance to Settlement and Elevation (Table C in S1 Appendix). If continuous variables are acting quadratically, model coefficients for the quadratic term should be negative, which indicates that although a leopard might select for a particular feature (e.g., elevation) or resource (e.g., dog abundance), it avoids areas where the feature is extreme or resource overabundant, resulting in a non-linear relationship [65].

The number of remote camera stations (40) dictated we keep the analysis simple according to Harrell’s rule, whereby each variable should have ~10 observations for meaningful interpretation [66,67]. As such, the most complex model contained only 4 independent variables. Due to the preponderance of 0s in the dataset, we utilized Generalized Linear Models (GLMs) with a Poisson distribution. Since this method requires the dependent variable to be integers, we could not use rates, so instead employed the total number of independent detections at each remote camera site (as detailed above) and incorporated an offset of the log of the number of 24-hr periods the cameras were active, into the models to ensure that survey effort was accounted for. For ease of interpretation and comparison we scaled all independent variables using the “scale()” function in R so that each had a mean of 0 and standard deviation of 1. The same set of models was used for all leopards combined as well as for male and female leopards separately, in order to understand sex-based variation in habitat suitability.

We determined Akaike’s Information Criterion for small sample sizes (AIC*c*) for each model and used ΔAIC*c* and model weights (*w*_*i*_) to rank models [58]. Model adequacy was determined using standard analyses of residuals (*i.e.,* residuals vs fitted values plots, normal Q-Q plots, scale-location plots, and residuals vs leverage plots). Model goodness-of-fit was determined using McFadden’s psuedo-R^2^ (1 – (residual deviance/ null deviance)).

### Leopard activity times

All photo-captures recorded the date and time of capture, and these times – after conversion to radians – were used to create fitted kernel density curves using default smoothing parameters in R package “overlap” [68]. A primary goal was to compare the activity patterns of leopards in the unprotected tea landscape to those in established protected areas in Sri Lanka, so we utilized existing remote camera data from Wilpattu National Park (WNP) from July to October 2015 [46] and Gal Oya National Park (GONP) from November 2017 – June 2020 (Table D in S1 Appendix). WNP is Sri Lanka’s largest PA (1317 km^2^), located in northwestern Sri Lanka’s arid and dry zones, whereas GONP (259 km^2^) is in the Intermediate zone and protects the watershed forest of Sri Lanka’s largest reservoir, the Senanayake Samudra. In both locations white flash remote cameras (Scoutguard in WNP; Cuddeback and Scoutguard in GONP) were set up along jeep tracks, walking paths and animal trails in a similar manner to the unprotected tea estate landscape. Camera height placement and settings were the same across sites. Leopard photo-captures for these sites were also used to create fitted kernel density curves as above.

In order to categorize leopard activity, the 24-hrs that characterize a full day were divided into discrete periods based on sunrise and sunset times in Sri Lanka [69] (Table E in S1 Appendix). Across the year sunrise ranged from 5:48–6:26 and sunset from 17:45–18:28. To determine crepuscular periods we therefore created a buffer of ~30 minutes of these minimum and maximum times, with diurnal and nocturnal periods therefore the remaining hours of light and dark, respectively, between crepuscular periods. Final time periods were defined as crepuscular (dawn) from 5:15–7:00, diurnal from 7:00–17:15, crepuscular (dusk) from 17:15–19:00, and nocturnal from 19:00–5:15.

To categorize leopard activity in the three locations we calculated Manly selectivity measures using ratios of use vs. availability for each time period [70]. The equation used was:


$$\begin{array}{c} w_{i}=o_{i}/\pi_{i} \end{array}$$

, where *w*_*i*_ is the selection ratio for the period *i*; *o*_*i*_ is the proportion of leopard trap events (photo-captures) in period *i*; and *π*_*i*_ is the proportion of length of time in period *i* to the length of time in all periods. When *w*_*i*_ > 1 the time period *i* is considered selectively used (*i.e.,* use > availability) and conversely, when *w*_*i*_ < 1 the time period *i* is considered avoided (*i.e.,* availability > use; [70–72].

To compare leopard activity patterns between the protected (WNP and GONP) and unprotected (tea estate landscape) areas, we compared the independent proportions of leopard observations for each of the time periods between the unprotected site and each protected site in turn. To do this we employed a two-sample, two-tailed Z-test [73].


$$\begin{array}{c} Z=(p_{\text{1}}-p_{\text{2}})\;/\;(p(\text{1}-p))/(\text{1}/n_{\text{1}}+\text{1}/n_{\text{2}}) \end{array}$$

, where p_1_ and p_2_ are the sample proportions, n_1_ and n_2_ are the sample sizes, and p is the total pooled proportion calculated as:


$$\begin{array}{c} p=(p_{\text{1}}n_{\text{1}}+p_{\text{2}}n_{\text{2}})/(n_{\text{1}}+n_{\text{2}}) \end{array}$$

We used p-values at a significance level of 0.05 to determine effect and whether the z-value was positive or negative to determine the direction of effect. We also calculated 95% confidence intervals (CI) for each effect with the interpretation that CIs that do not overlap 0 represent strong effects.

## Results

Across 1258 24-hour remote camera periods we recorded 90 separate leopard detections, 94.4% of which were definitively individually identified (N = 85). Three leopard images were not clear enough to determine age/sex or individual ID, while 2 additional photo captures of adult males could not be individually identified with certainty. These images were discarded from the density analysis, as were the two cub images, resulting in 83 leopard photo-captures used for density analysis. The leopard site use analysis used all 90 leopard images, with 54 photo-captures of adult males used for the “male only” site use analysis and 31 photo-captures of adult females used for the “female only” site use analysis. In total, 18 individual leopards were identified, 16 mature individuals (6 M, 10 F) and 2 cubs. Leopards were photo-captured at 26 (65%) camera stations.

### Leopard density

The top-ranked model (AIC*cwt* = 0.904) for estimating leopard density allowed for heterogeneity in the spatial parameter related to home range size (*σ ~ h2*; Table 1). Density estimates stabilized with a buffer width of 11000m (S2 Table in S1 Appendix), so employing the above hybrid-mix model formulation with varying effort and 11000m buffer, we estimated the study area’s mature leopard population density as 8.82 ± SE 2.42 leopards/100 km^2^ (95% CI = 5.21–14.95). Adult sex ratio (ASR) of observed individuals (37.5%; 1M: 1.67F) was considerably different from that determined by the hybrid mixture model (*pmix*) (16.1%; 1M: 5.17F; Table 2). The probability of detection at home range centre (*g0*) was 0.046 ± SE 0.008 for both sexes, whereas the spatial parameter (*σ*) was 2409m ± SE 240 for males and 737m ± SE 87 for females (Table 2).

**Table 1:** Comparison of spatially explicit capture-recapture (R package *secr*) model selection parameters for 2-class hybrid mixture models (*hcov* in R).

Model	Description	K^a^	LogLik	AIC*c*^b^	ΔAIC*c*^c^	AIC*cwt*^d^
*g0 ~ 1, σ ~ h2*	Hybrid mixed model, variation between sexes affecting *σ*	5	−152.146	320.291	0	0.904
*g0 ~ h2, σ ~ h2*	Hybrid mixed model, variation between sexes affecting *g0* and *σ*	6	−151.717	324.768	4.477	0.096
*g0 ~ h2, σ ~ 1*	Hybrid mixed model, variation between sexes affecting *g0*	5	−166.834	349.669	29.378	0
*g0 ~ 1, σ ~ 1*	No variation	4	−174.903	361.443	41.152	0

Hybrid mixture models allow sex to be modeled as a covariate with g0 = the probability of capture at home range center and σ = spatial parameter related to home range size. ^**a**^Number of model parameters ^**b**^Akaike Information Criterion, adjusted for small sample size ^**c**^Difference from best ranking (lowest AICc) model ^**d**^Model weighting.

**Table 2:** Parameters estimated by the best spatially explicit capture-recapture (R package *secr*) model for leopard density estimation (g0 ~ 1, *σ ~ h2*).

	Mean±SE		95% CI	
Parameter^a^	♂	♀	♂	♀
***g0***	0.046 ± 0.008	0.046 ± 0.008	0.032 - 0.065	0.032 - 0.065
***σ***	2409 ± 240.1	737 ± 87.0	1983 - 2927	585 - 928
***pmix***	0.161 ± 0.073	0.839 ± 0.073	0.063 - 0.357	0.643 - 0.937

^a^*g0* = probability of capture at home range centre; *σ =* spatial parameter (in meters) related to home range size; *pmix* = sex ratio.

The observed density was lower than what has been found in Sri Lanka’s best protected National Parks, but in almost every case 95% Cis overlap, indicating that this difference is not significant (Table 3).

**Table 3:** Leopard density (mature individuals/100 km^2^) comparison across published Sri Lankan studies that employed closed population, spatially explicit capture-recapture (R package *secr*) methods.

			Density Estimate	95% Density Range
**Authors (year published)**	**Location**	**Habitat type**	**mature individuals/100 km²**	**mature individuals/100 km²**
Kittle, Watson & Fernando (2017)	Yala NP (Block 1)	Woody scrub	12.1	(10 - 14.7)
Kittle & Watson (2017)	Horton Plains NP	Montane forest and patna grassland	11.7	(0.9 - 22.5)
Webb *et al.* (2020)	Horton Plains NP	Montane forest and patna grassland	10.6 & 15.1	(8 - 16 & 13.3 −18.7)
Kittle, Watson & Samaranayake (2021)	Wilpattu NP	Dry evergreen monsoon forest	10.4	(7.3 - 14.9)
Samarasinghe *et al*. (2022)	Wilpattu NP	Dry evergreen monsoon forest	18	(16 - 21)
This study	Unprotected tea estates	Tea, Eucalyptus, Pinus, sub-montane patch forest mosaic	8.8	(5.2 - 15)

### Leopard site utilization

The model that best explained overall leopard site utilization was a co-existence model that included both human and dog RAI as well as their quadratic terms (Table 4). The coefficients for both quadratic terms were negative which is the only biologically plausible option [64] (Table F in S1 Appendix) and indicates that leopard site use was maximized at intermediate levels of both human and dog abundance (*i.e.,* both areas where human and dog occurrence were high and where human and dog occurrence were low, showed lower levels of leopard use). Model *R²* = 0.26.

**Table 4:** Comparison of all site utilization GLM models.

		All Leopards				Male Leopards			Female Leopards		
Model Type	Model	k	AICc	∆AICc	*wi*	AICc	∆AICc	*wi*	AICc	∆AICc	*wi*
Co-existence	Human_RAI	1	190.360	16.72	0.00018	142.999	10.34	0.00473	119.205	8.61	0.00557
Co-existence	Human_RAI_quadratic	2	176.787	3.15	0.16327	135.684	3.03	0.18327	116.245	5.65	0.02446
Co-existence	Dog_RAI	1	192.067	18.43	0.00008	142.723	10.07	0.00543	120.340	9.74	0.00316
Co-existence	Dog_RAI_quadratic	2	178.750	5.11	0.06118	138.349	5.69	0.04835	113.480	2.88	0.09749
Co-existence	Human_Dog_RAI_quadratic	4	**173.636**	**0.00**	**0.78910**	137.864	5.21	0.06163	113.750	3.15	0.08514
Co-existence	Distance_Settlement	1	193.048	19.41	0.00005	142.805	10.15	0.00521	120.557	9.96	0.00283
Co-existence	Distance_Settlement_quadratic	2	183.367	9.73	0.00608	**132.656**	**0.00**	**0.83292**	121.557	10.96	0.00172
Co-existence	Distance_Road	1	193.991	20.36	0.00003	144.581	11.92	0.00214	120.520	9.92	0.00288
Habitat suitability	Distance_PA	1	192.326	18.69	0.00007	143.290	10.63	0.00409	120.504	9.90	0.00291
Habitat suitability	Distance_Forest	1	194.396	20.76	0.00002	145.029	12.37	0.00171	120.534	9.93	0.00286
Landscape	Elevation	1	193.269	19.63	0.00004	144.732	12.08	0.00199	116.540	5.94	0.02111
Landscape	Elevation_quadratic	2	195.482	21.85	0.00001	146.897	14.24	0.00067	118.326	7.73	0.00864
Prey	Black-naped_hare_RAI	1	191.450	17.81	0.00011	144.722	12.07	0.00200	***112.607***	***2.01***	***0.15084***
Prey	Muntjak_RAI	1	192.780	19.14	0.00005	144.443	11.79	0.00230	***112.265***	***1.67***	***0.17893***
Prey	Black-naped_hare_Muntjak_RAI	2	193.258	19.62	0.00004	146.669	14.01	0.00075	**110.600**	**0.00**	**0.41145**
Null	Null	0	192.192	18.56	0.00007	142.867	10.21	0.00505	118.340	7.74	0.00858

Top models are in bold based on the lowest Akaike Information Criterion for small sample size values (AICc). If the difference between AICc values for the best model and a second model (∆AICc) is < 2, those models are considered equivalent. Model weights (*w*_*i*_) are also shown.

Male leopard site utilization was again best explained by a co-existence model, this time the model incorporating both the distance to human settlement and its quadratic term (Table 4). Again, the quadratic term co-efficient was negative suggesting a preference for intermediate distance from human settlements (Table F in S1 Appendix). Model *R²* = 0.19.

Female leopard site utilization was best explained by a prey model that incorporated both black-naped hare and muntjak RAI (Table 4). Both co-efficients were strongly positive indicating that female leopards were more likely to use sites with high occurrence of these prey species (Table F in S1 Appendix). Model *R²* = 0.16.

### Leopard activity times

**Fig 2:**
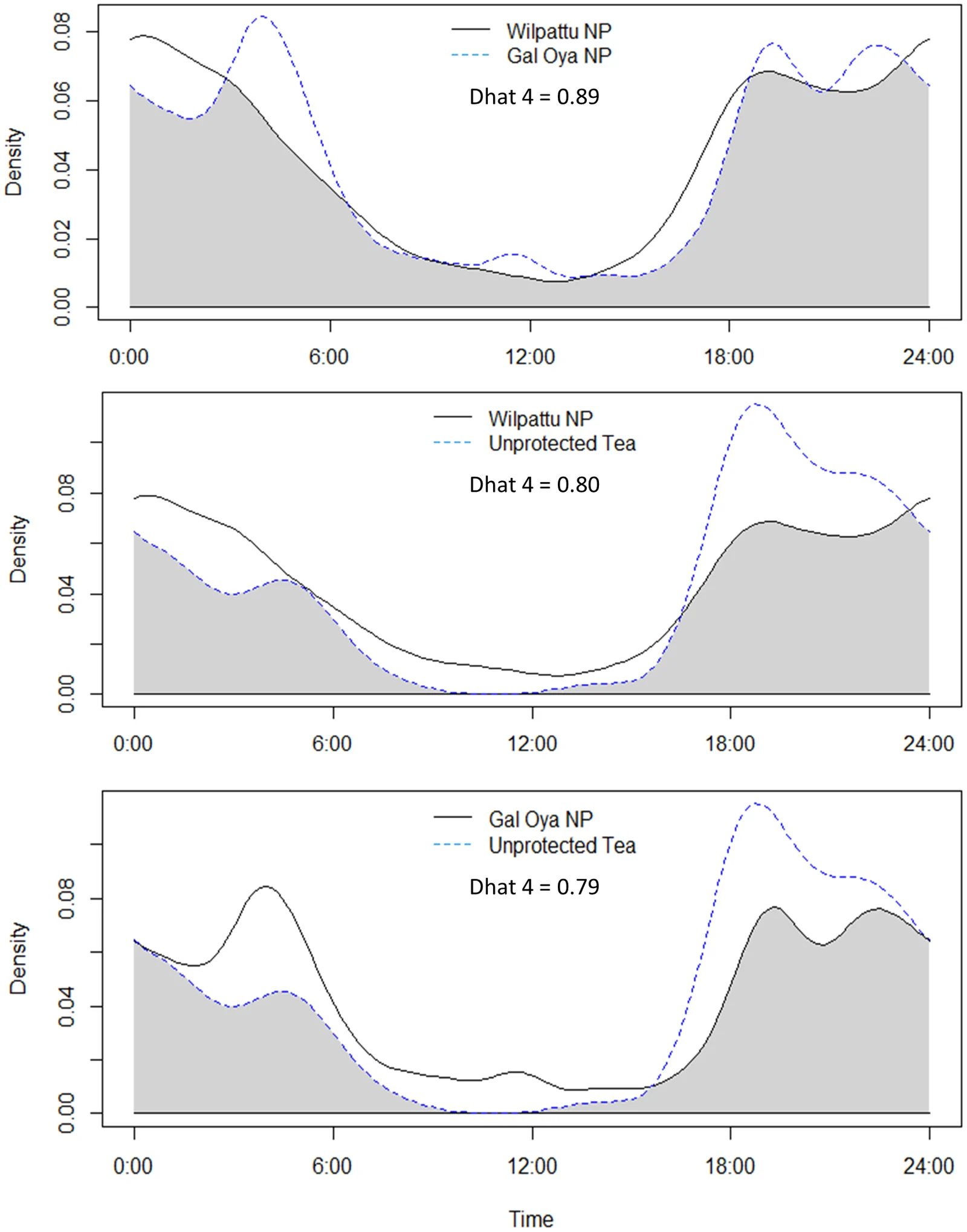
The fitted kernel density curves using default smoothing parameters (R package “overlap”) comparing two Sri Lankan Protected Areas (Wilpattu National Park and Gal Oya National Park; top), the unprotected tea estate landscape (this study) and Wilpattu NP (middle), and the unprotected tea estate landscape (this study) with Gal Oya NP (bottom). While leopards in all three study areas would be described as primarily active at dusk and nocturnally, the unprotected tea estate landscape leopards were significantly less active diurnally and more active at dusk than the two NP leopards.

A total of 90 independent leopard observations were utilized for determining activity patterns from the unprotected tea estate landscape, with 183 observations from WNP [46] and 214 from GONP (Table D in S1 Appendix). All three leopard populations were characterized as being active at dusk and nocturnally, with low diurnal activity (Table 5). Inter-site comparisons showed that there was no difference between the two protected areas, but leopards in the unprotected tea landscape were significantly less diurnal and more active around dusk than the protected area landscapes (Fig 2; Table 6).

**Table 5:** Activity period breakdown of leopards across three Sri Lankan study sites (2 National Parks and one unprotected landscape) based on remote camera photo-captures.

	Cr(Dawn)		Diurnal		Cr(Dusk)		Nocturnal		
	5:15 − 7:00		7:00 - 17:15		17:15 - 19:00		19:00 - 5:15		
Location	% Obs	*w*_*i*_	% Obs	*w*_*i*_	% Obs	*w*_*i*_	% Obs	*w*_*i*_	Category
Wilpattu NP	6.0	0.82	14.2	0.33	11.5	1.57	68.3	1.60	Cr(Du)/N
Gal Oya NP	6.0	0.83	13.0	0.30	8.8	1.21	72.1	1.69	Cr(Du)/N
Unprotected Tea	4.4	0.61	4.4	0.10	23.3	3.20	67.8	1.59	Cr(Du)/N

Activity periods are Crepuscular either around dawn (Cr(Dawn)) or dusk (Cr(Dusk)), Diurnal and Nocturnal. The proportion of total observations during each period is shown (% Obs) as well as the Manly selection index (*w*_*i*_). When *w*_*i*_ > 1 selection for the time period is indicated, whereas when *w*_*i*_ < 1 avoidance is indicated, with greater selection or avoidance suggested as *w*_*i*_ approaches 2 or 0 respectively.

**Table 6:** The comparison of independent proportions of leopard observations for each of the time periods (Diurnal, Nocturnal and Crepuscular dawn and dusk) between the unprotected tea landscape and the two National Parks (Wilpattu NP and Gal Oya NP).

		Wilpattu NP			Gal Oya NP		
		z-score	P-value	95% CI	z-score	P-value	95% CI
**Unprotected Tea**	**Cr(Dawn)**	−0.5464	0.5848	(−0.0706, 0.0386)	−0.5577	0.5771	(−0.0689, 0.0369)
	**Diurnal**	*−2.4358*	*0.0149*	*(−0.1584, −0.0276)*	*−2.2381*	*0.0252*	*(−0.1478, −0.0242)*
	**Cr(Dusk)**	*2.5399*	*0.0111*	*(0.0192, 0.2168)*	*3.4254*	*0.0006*	*(0.0498, 0.2402)*
	**Nocturnal**	−0.0833	0.9334	(−0.1227, 0.1127)	−0.7535	0.4512	(−0.1566, 0.0706)

Two-sample, two-tailed Z-tests were employed with z-scores, P-values and 95% Confidence Intervals shown. When 95%CI do not overlap 0 it is considered strong evidence of difference between sites and is indicated here by italics.

## Discussion

### Leopard density

Leopard density in this unprotected, tea-estate dominated landscape was lower than what has been documented in three of Sri Lanka’s iconic, best protected National Parks – Yala, Wilpattu and Horton Plains – but this difference was far less stark than anticipated, with 95% CIs showing considerable overlap. Although this result does not imply ecological equivalence between PAs and unprotected landscapes, it does suggest that protected areas might not automatically provide more suitable habitat for large carnivores, and echoes results from Finland where the population densities of three of the four carnivore species in a nation-wide study – lynx (*Lynx lynx*), wolf (*Canis lupus*) and wolverine (*Gulo gulo*) – were unaffected by PA status [74]. These results suggest that the unprotected tea estate landscape, at least in this part of the Central Highlands, provides reasonable habitat suitability for the Sri Lankan leopard. This is likely due to several factors, including an available natural prey base [34,62], a rugged landscape that allows for refuge areas relatively inaccessible to humans [75], and the fact that the main crop being cultivated (tea) is a low, dense shrub that can provide good cover for movement [76]. Furthermore, although humans are widespread on the landscape and are active in the day, at nightfall people largely retreat back to their clustered communities leaving much of the region both dark and devoid of human presence. That leopards are renowned for their adaptability in human-dominated areas anyway [77], means that these key factors may be sufficient to allow a robust population here.

It is important to note that more recent surveys of protected area leopard populations in Sri Lanka have documented very high densities: 54.6/100 km^2^ in Yala NP Block 1 [78] and 40/100 km^2^ in neighbouring Kumana NP [79]. However, one study did not use *secr* methodology [78] while neither density estimate complied with closed population assumptions [78,79], so we felt unable to compare these with studies using more standard methodology (Table 3). However, if these recent estimates are accurate it represents a substantial population increase, the underlying causes of which require further investigation. One possibility is that these NPs are increasingly important refuges due to increased habitat loss and associated anthropogenic pressures beyond PA boundaries [80]. Previous research in WNP has detected evidence of avoidance of park boundaries by leopards, increased intra-specific competition [46] and potential inbreeding [56], which collectively suggests a population hemmed in by external forces.

While the adult sex ratio (ASR) of direct remote camera observations was female biased (37.5% M), this bias was stronger (16.1% M) using the secr hybrid mixture model which estimates detection bias and sex-specific movement. This model allowed for variation between sexes to influence the spatial parameter related to home range size (σ), but not the probability of capture at home range centre (g0). The much larger σ seen for males compared to females (x̅ = 2409 vs 737) indicates substantial home range size variation resulting in an even more female-biased ASR than was apparent from raw observations, as the latter would have likely included observations of males far from home range centres. Female-biased ASRs have been detected regularly in large felids given that most – including leopards – are polygynous with one male range overlapping the ranges of several females [81]. Larger study areas have been linked to increased female-biased ASR in both medium (~50 kg) and large felids (> 100 kg) felids [82]. Although the current study area is not a bounded PA, the remote camera survey was representative of an extensive landscape - ~ 2,400 km^2^ [34] – of broadly similar composition (*i.e.,* a mosaic of tea cultivation, forest patches, plantation forests and communities), which aligns with the expectations of a female biased ASR.

Higher population density in large study areas has been similarly linked to increased female-biased ASR [82]. Male leopards have been shown to undergo density-dependent dispersal, due to increased competition and higher rates of male emigration [83]. Concurrently, when population density increases, female leopard range size decreases while male range size remains stable, resulting in a female-biased ASR [84]. The current survey described a surprisingly high adult leopard density which, taken together with the landscape’s relatively broad extent, again aligns with expectations of female-biased ASR [82].

### Leopard site utilization

Leopards in the unprotected Central Highlands do not appear to be spatially avoiding humans, but to be highly cognizant of their presence, a factor which they seem to incorporate into space use decisions. This is consistent with the landscape of co-existence concept [85] which sees predators alter spatio-temporal patterns in order to maximize the probability of ensuring long-term co-existence with humans [86]. Leopards were most likely to be found at intermediate levels of human use, which suggests an avoidance of the most high-use – and therefore high-risk – areas [87] tempered by the ability to tolerate general human presence [11]. Eurasian lynx in Norway were similarly found to favour areas of intermediate human disturbance, as well as intermediate preferred prey abundance, apparently trading off the use of more prey rich areas with the avoidance of higher, human-induced risk [88]. In the Swiss Alps, lynx have been shown to employ additional behavioral adaptations to enact this tradeoff, by visiting prey rich but high human use, high-risk areas at night and moving more rapidly when in proximity to areas of high human disturbance [86]. While these temporal shifts are consistent with broad patterns seen in this study, additional data would be required to determine whether leopards in this system similarly alter movement rates when close to human disturbance or high human use areas.

Leopards were also more likely to be found at intermediate levels of domestic dog use here which may also be reflecting the perception by leopards of a landscape of co-existence [85]. Dogs are unlikely to be a direct threat to leopards in this landscape – except potentially to small cubs – as most dogs are relatively small (~ 15 kg) and don’t form packs here, so avoidance by leopards of high dog-use areas is unlikely due solely to the presence of dogs. Additionally, although dogs are a prey source in this landscape, they comprise < 10% of leopard diet and are not preferentially selected [62], so it is also unlikely that leopards make land-use decisions heavily influenced by the perception of dogs as prey, unlike in parts of India where wild prey is limited or absent [11,89,90]. Instead, while their use of the landscape is not highly correlated with human use (|r| < 0.7), dogs remain closely associated with people, and it may be this association which results in the similar space-use tradeoffs observed with human use areas. Male leopards were seen to avoid human settlements, perhaps due to a perception of greater risk in proximity to humans [91]. Leopards within PAs have been observed to avoid PA boundaries in Sri Lanka [46] and Thailand [92] when those boundaries represent high levels of human activity. Female leopards were more likely to be found in areas of high natural prey availability, consistent with this being a key factor underlying the size and location of female leopard home ranges [55,93]. Red muntjac are almost the ideal size of highly preferred leopard prey [63], while the smaller black-naped hare (~2.5 kg) are the most frequently predated species in the study area [62] and have very high levels of activity overlap with leopards here. Similar sized prey species, including the yellow-striped chevrotain (*Moschiola kathygre*) and even domestic dogs in this landscape, may be especially important prey species at key times for female leopards, as they regularly need to transport prey to den sites and/or dependent cubs [94,95].

It is important to recognize that the R^2^ values of the top site utilization models were modest (0.16–0.26) which suggests that influential variables were not included in the modelling procedure. Ecological systems are complex with numerous environmental, anthropogenic, physiological and social factors that influence how predators utilize their habitat and even key factors can be overlooked or unmeasured. For example, social interactions between leopards such as mating and cub-rearing can alter space use decisions in both the short- and longer term [96,97], while seasonal climatic factors can impact site use on a cyclical basis [98]. As such, it is important to build on studies such as this one in an attempt to reach a more holistic understanding of leopard space use decisions and their underlying factors.

### Leopard activity times

Wildlife in human-dominated landscapes often alter their behavior in order to avoid confrontations with people, which is typically expressed through increased nocturnal activity [28]. Here leopards showed similar general behavioral patterns as those within Sri Lankan PAs, in being predominantly active nocturnally and at dusk, but in contrast, their daytime activity was almost negligible, with compensation made at nightfall when a sharp spike in activity was observed. This increased activity around sunset suggests a strategy of “lying low” during the day to avoid diurnally active people [92], with a subsequently increased need to move as soon as daylight subsides. In unprotected areas outside Nepal’s Chitwan National Park, where daytime natural resource collection by people is high, temporal avoidance of humans was also especially pronounced [99]. It is important to note that there was not perfect overlap regarding the time of year monitored for all 3 sites; that GONP was monitored for considerably longer than both WNP and the tea estate landscape (20 months vs 4 months); and that the sites are all located within different climatic zones. As such, there may be additional factors which partially underlie these observations. However, the strong similarities in activity patterns detected between WNP and GONP, despite having varied monitoring periods and being in different climatic zones, supports the conclusion that leopards in the tea estate landscapes are indeed acting differently and employing temporal partitioning to avoid people.

This temporal partitioning is likely to be complemented by fine-scale spatial partitioning during periods of human activity. In Sri Lanka’s tea-estate dominated Central Highlands, the landscape is a mosaic of land use types with vast expanses of tea cultivation interspersed with pockets of natural forest, plantation forest and scrub/shrub areas where previous tea cultivation has been discontinued. It is in these areas that leopards appear to spend the daytime hours before emerging to roam the extensive network of tea estate roads and walking paths that traverse the landscape and which are used by estate workers and community members during the day. Cultivated tea fields can provide adequate cover for leopards to move through, but given the regular daytime presence of estate workers in these areas and the relatively low frequency with which leopards are sighted here, it is unlikely that they are extensively used as daytime resting places. However, small cubs have been found on several occasions within the tea bushes, left there by temporarily absent mothers, which suggests that leopards do sometimes utilize the tea fields in this way.

Although temporal partitioning can be an effective way to ensure co-existence in shared landscapes (e.g., [100]) particularly for persecuted species or predators [101], wire snares, which remain on the landscape even when people have left, transcend this adaptation by de-coupling the threat from the presence of humans. This threat is particularly prevalent in Sri Lanka’s unprotected Central Highlands and is the leading known cause of leopard mortality in this region [35]. Widespread snaring can have a profound impact on mammalian biodiversity [102,103], including apex predators whether they are targeted [104] or not [105]. While snares in the Central Highlands of Sri Lanka typically are not set to target leopards, being unselective they nevertheless take a toll [35] and provide a stark reminder that behavioural adaptations by wildlife are not, on their own, sufficient to offset human-induced mortality. In order to ensure the long-term viability of leopards in this unprotected landscape it is essential to curb the use of wire snares.

## Conclusion

This unprotected, hill country leopard population exhibits a density lower than Sri Lanka’s PAs for which comparable data are available, however the difference was considerably smaller than anticipated. Furthermore, the 3 compared PAs – Yala (Block 1), Wilpattu and Horton Plains – are Sri Lanka’s most iconic National Parks, all of which are well known as leopard “hotspots”. This suggests that the tea landscape of the southern Central Highlands is conducive for leopard presence despite the widespread and high level of human activity found here. Spatial partitioning is not how this relatively robust leopard population negotiates potential anthropogenic impacts, although results suggest that leopards here do consider human presence when making space use decisions. Instead, it appears that temporal partitioning, with decreased leopard activity in the day compensated by increased activity around dusk, is the behavioural adaptation which underlies human-leopard co-existence here.

To summarize the findings of what is the first comprehensive study of leopard density and behaviour outside of the island’s protected areas, leopards appear to have adapted well to this unprotected, human-dominated landscape with temporal partitioning a clear behavioral adaptation undertaken by them to reduce direct human threats and essentially foster human-leopard co-existence. From a management perspective, to ensure that the island’s leopard population remains robust in the long term, the leopards in this unique, vulnerable region of Sri Lanka require ongoing monitoring and to be subject to conservation initiatives aimed at ensuring their long term viability. These include the maintenance of existing habitat patches and corridors that allow, at least, for the present level of connectivity. Key to the implementation of conservation initiatives is the need to maintain or strengthen present levels of human-leopard co-existence. To do this it is imperative to 1) maintain current temporal patterns (limited nighttime use by humans) to avoid off-setting leopard behavioral adaptions, 2) ensure that the region’s sizeable natural prey base remains stable as this keeps leopards from switching to a diet more reliant on domestic species, and 3) minimize – or ideally, eliminate – the use of snares in this region, as these can transcend behavioural adaptations and render this leopard population highly vulnerable.

## Supporting information

S1 Fig Sri Lankan leopard in tea estate landscape. (TIF)

S1 Appendix Supporting Tables. Table A contains the remote camera location coordinates and running durations for this study. Table B contains the remote camera array buffer estimation. Table C contains the final spatial utilization model variables. Table D contains the remote camera location coordinates and running durations for Gal Oya NP. Table E contains the monthly sunrise and sunset times for the study area. Table F contains the coefficient values, standard errors, z-values and P-values for variables in all top spatial models.
(DOCX)
