# Supplementary material for: Same place, different time: Leopards (Panthera pardus kotiya) employ temporal partitioning as a co-existence mechanism in a human-dominated, unprotected agricultural landscape in Sri Lanka
Source: PLoS One. 2026 Sep 9;21(9):e0335296. doi: 10.1371/journal.pone.0335296 (PMC13557350; doi:10.1371/journal.pone.0335296)
Supplement: Supplementary file 1 [file pone.0335296.s002.docx]

**S1 Appendix: Supporting Tables**

Table A: Remote camera location coordinates and running durations for this study.

| **#** | **Round** | **Trap ID** | | **N** | **E** | **Start date** | **End date** | **Days** | **camera#** | **Elevation** |
| --- | --- | --- | --- | --- | --- | --- | --- | --- | --- | --- |
| 1 | 1 | A1 | 6.85555 | | 80.57017 | 12/08/2016 | 15/9/2016 | 34 | 1 | 1417 |
| 2 | 1 | A2 | 6.85403 | | 80.56313 | 12/08/2016 | 15/9/2016 | 34 | 1 | 1514 |
| 3 | 1 | A3 | 6.79506 | | 80.63971 | 13/8/2016 | 16/9/2016 | 34 | 1 | 1689 |
| 4 | 1 | A4 | 6.80469 | | 80.63348 | 13/8/2016 | 16/9/2016 | 34 | 1 | 1530 |
| 5 | 1 | A5 | 6.80130 | | 80.63537 | 31/8/2016 | 16/9/2016 | 16 | 2 | 1593 |
| 6 | 1 | A6 | 6.80843 | | 80.62398 | 13/8/2016 | 15/9/2016 | 33 | 2 | 1530 |
| 7 | 1 | A7 | 6.83432 | | 80.60194 | 13/8/2016 | 15/9/2016 | 33 | 2 | 1341 |
| 8 | 1 | A8 | 6.78985 | | 80.63635 | 14/8/2016 | 14/9/2016 | 31 | 2 | 1703 |
| 9 | 1 | A9 | 6.82314 | | 80.60997 | 14/8/2016 | 15/9/2016 | 32 | 2 | 1399 |
| 10 | 1 | A10 | 6.76416 | | 80.62798 | 15/8/2016 | 14/9/2016 | 30 | 2 | 1543 |
| 11 | 1 | A11 | 6.80367 | | 80.60770 | 15/8/2016 | 14/9/2016 | 30 | 1 | 1320 |
| 12 | 1 | A12 | 6.83804 | | 80.58246 | 15/8/2016 | 14/9/2016 | 30 | 2 | 1355 |
| 13 | 2 | B1 | 6.79008 | | 80.61356 | 17/9/2016 | 21/10/2016 | 34 | 1 | 1349 |
| 14 | 2 | B2 | 6.76789 | | 80.60482 | 17/9/2016 | 20/10/2016 | 33 | 1 | 1516 |
| 15 | 2 | B3 | 6.77198 | | 80.58645 | 17/9/2016 | 20/10/2016 | 33 | 2 | 1491 |
| 16 | 2 | B4 | 6.77945 | | 80.58521 | 17/9/2016 | 20/10/2016 | 33 | 1 | 1569 |
| 17 | 2 | B5 | 6.79688 | | 80.57754 | 17/9/2016 | 20/10/2016 | 33 | 2 | 1476 |
| 18 | 2 | B6 | 6.82264 | | 80.58069 | 17/9/2016 | 20/10/2016 | 33 | 1 | 1242 |
| 19 | 2 | B7 | 6.80127 | | 80.56697 | 18/9/2016 | 20/10/2016 | 32 | 2 | 1394 |
| 20 | 2 | B8 | 6.77895 | | 80.55950 | 18/9/2016 | 20/10/2016 | 32 | 1 | 1652 |
| 21 | 2 | B9 | 6.77784 | | 80.55844 | 18/9/2016 | 20/10/2016 | 32 | 1 | 1724 |
| 22 | 2 | B10 | 6.79534 | | 80.54693 | 18/9/2016 | 20/10/2016 | 32 | 1 | 1434 |
| 23 | 2 | B11 | 6.79027 | | 80.59930 | 19/9/2016 | 21/10/2016 | 32 | 2 | 1433 |
| 24 | 2 | B12 | 6.79227 | | 80.60082 | 19/9/2016 | 21/10/2016 | 32 | 1 | 1423 |
| 25 | 2 | B13 | 6.82267 | | 80.59392 | 20/9/2016 | 20/10/2016 | 30 | 2 | 1360 |
| 26 | 2 | B14 | 6.85693 | | 80.56776 | 10/05/2016 | 17/10/2016 | 12 | 1 | 1458 |
| 27 | 3 | C1 | 6.87110 | | 80.55450 | 22/10/2016 | 26/11/2016 | 35 | 2 | 1359 |
| 28 | 3 | C2 | 6.88532 | | 80.54305 | 22/10/2016 | 26/11/2016 | 35 | 2 | 1136 |
| 29 | 3 | C3 | 6.88836 | | 80.54225 | 22/10/2106 | 26/11/2016 | 35 | 1 | 1066 |
| 30 | 3 | C4 | 6.80834 | | 80.52816 | 23/10/2016 | 27/11/2016 | 35 | 2 | 1637 |
| 31 | 3 | C5 | 6.82354 | | 80.51061 | 23/10/2016 | 27/11/2016 | 35 | 2 | 1355 |
| 32 | 3 | C6 | 6.84139 | | 80.51896 | 23/10/2016 | 27/11/2016 | 35 | 2 | 1619 |
| 33 | 3 | C7 | 6.83881 | | 80.52414 | 23/10/2016 | 27/11/2016 | 35 | 2 | 1622 |
| 34 | 3 | C8 | 6.87635 | | 80.53020 | 24/10/2016 | 26/11/2016 | 33 | 1 | 1110 |
| 35 | 3 | C9 | 6.88060 | | 80.52334 | 24/10/2016 | 26/11/2016 | 33 | 2 | 942 |
| 36 | 3 | C10 | 6.84184 | | 80.53729 | 24/10/2016 | 27/11/2016 | 34 | 2 | 1371 |
| 37 | 3 | C11 | 6.81728 | | 80.54379 | 24/10/2016 | 27/11/2016 | 34 | 1 | 1249 |
| 38 | 3 | C12 | 6.86207 | | 80.54788 | 25/10/2016 | 26/11/2016 | 32 | 1 | 1303 |
| 39 | 3 | C13 | 6.84476 | | 80.57401 | 25/10/2016 | 26/11/2016 | 32 | 1 | 1376 |
| 40 | 3 | C14 | 6.80688 | | 80.59595 | 11/11/2016 | 04/12/2016 | 15 | 1 | 1317 |

Table B: Remote camera array buffer estimation.

The R “secr” package was used to determine the buffer size, first employing secr’s “suggest.buffer” function to determine a suitable starting width (7601m) for the concave polygon buffer around the detector array. The “make.mask” function ws then used to create a set of habitat masks starting with a smaller buffer (4000m) and sequentially increasing its width, by 1000m, around the trapping grid, up to 15000m. To ensure that individuals outside the buffer area cannot be captured within and artificially inflate density estimates, the “check.mask” function was then utilized to compare models using the 12 mask size iterations until model LogLikelihood estimates stabilized at 3 decimal places (Table S2) [48,51] and utilized this buffer size for further analysis.

| **Buffer (m)** | **esa** | **LogLik** | **D-hat** | **se(D-hat)** |
| --- | --- | --- | --- | --- |
| 4000 | 30251.56 | -165.9761 | 0.000528896 | 0.000134708 |
| 5000 | 35179.66 | -164.4858 | 0.000454807 | 0.000116905 |
| 6000 | 38181.94 | -164.4024 | 0.000419045 | 0.000110465 |
| 7000 | 38981.96 | -164.5351 | 0.000410445 | 0.000110442 |
| 8000 | 39068.91 | -164.5834 | 0.000409531 | 0.000110768 |
| 9000 | 39074.67 | -164.5931 | 0.000409471 | 0.000110860 |
| 10000 | 39074.04 | -164.5946 | 0.000409478 | 0.000110879 |
| **11000** | **39073.71** | **-164.5948** | **0.000409481** | **0.000110882** |
| 12000 | 39073.64 | -164.5949 | 0.000409482 | 0.000110883 |
| 13000 | 39073.63 | -164.5949 | 0.000409482 | 0.000110883 |
| 14000 | 39073.63 | -164.5949 | 0.000409482 | 0.000110883 |
| 15000 | 39073.63 | -164.5949 | 0.000409482 | 0.000110883 |

Table C: Final spatial utilization model variables.

| **Underlying Factor** | **Variable** | **Variable Name** | **Range** | **Detail** |
| --- | --- | --- | --- | --- |
| Co-existence | Relative abundance of humans | Human_RAI | 0 - 2046.88 | (# of detections of individual humans and vehicles) / # remove camera 24-hr surveillance periods)*100 |
| Co-existence | Relative abundance of domestic dogs | Dog_RAI | 0 - 306.25 | (# of detections of individual domestic dogs) / # remove camera 24-hr surveillance periods)*100 |
| Co-existence | Human infrastructure - settlements | Distance_Settlement | 120 - 1092 | Euclidean distance (m) from remote camera location to nearest human settlement |
| Co-existence | Human infrastructure - roads | Distance_Road | 10 - 4978 | Euclidean distance (m) from remote camera location to nearest primary or secondary road |
| Habitat suitability | Protected Areas | Distance_PA | 0 - 4965 | Euclidean distance (m) from remote camera location to nearest protected area |
| Habitat suitability | Forest patches | Distance_Forest | 0 - 1604 | Euclidean distance (m) from remote camera location to nearest forest patch |
| Landscape | Elevation | Elevation | 942 - 1724 | Elevation above sea level (m) taken from GPS at each remote camera location |
| Prey | Relative abundance of black-naped hare | Black-naped_hare_RAI | 0 - 108.57 | (# of detections of individual black-naped hares) / # remove camera 24-hr surveillance periods)*100 |
| Prey | Relative abundance of red muntjak | Muntjak_RAI | 0 - 27.27 | (# of detections of individual red muntjak) / # remove camera 24-hr surveillance periods)*100 |

Table D: Remote camera location coordinates and running durations for Gal Oya NP.

|  | **Location** | | **Camera duration** | | **Days** | **Leopard** |
| --- | --- | --- | --- | --- | --- | --- |
| **Trap ID** | **N** | **E** | **Start date** | **End date** | **active** | **Occasions** |
| Go_Mull1 | 7.28885 | 81.44673 | 04/11/2017 | 11/02/2018 | 31 | 3 |
| Go_Mull1b | 7.29057 | 81.44527 | 11/01/2019 | 23/10/2019 | 220 | 27 |
| Go_Mull2 | 7.31291 | 81.45449 | 04/11/2017 | 23/10/2019 | 486 | 45 |
| Go_Mull3 | 7.33287 | 81.45532 | 04/11/2017 | 23/10/2019 | 578 | 17 |
| Go_Nam7 | 7.24934 | 81.47696 | 12/01/2019 | 11/07/2019 | 115 | 5 |
| Go_Nam8 | 7.25806 | 81.49095 | 12/01/2019 | 11/07/2019 | 120 | 3 |
| Go_Nil1 | 7.19142 | 81.42228 | 11/08/2019 | 11/06/2020 | 279 | 6 |
| Go_Nil2 | 7.19325 | 81.41563 | 11/08/2019 | 11/06/2020 | 192 | 27 |
| Go_Nil3 | 7.18501 | 81.40483 | 11/08/2019 | 11/06/2020 | 236 | 19 |
| Go_Nil4 | 7.18999 | 81.38432 | 11/08/2019 | 11/06/2020 | 304 | 36 |
| GO_Nil5 | 7.20445 | 81.37038 | 11/08/2019 | 11/06/2020 | 304 | 16 |
| GO_Nil6 | 7.18143 | 81.39923 | 24/10/2019 | 11/06/2020 | 213 | 1 |
| GO_Nil7 | 7.19737 | 81.37716 | 24/10/2019 | 11/06/2020 | 200 | 9 |
| **TOTAL** |  |  |  |  | **3278** | **214** |

Table E: Monthly sunrise and sunset times for the study area.

|  | **Sunrise** | | **Sunset** | |
| --- | --- | --- | --- | --- |
| **Month** | **Earliest** | **Latest** | **Earliest** | **Latest** |
| January | 06:19 | 06:26 | 18:01 | 18:14 |
| February | 06:20 | 06:26 | 18:14 | 18:18 |
| March | 06:05 | 06:20 | 18:16 | 18:18 |
| April | 05:52 | 06:05 | 18:15 | 18:16 |
| May | 05:48 | 05:52 | 18:15 | 18:20 |
| June | 05:48 | 05:51 | 18:21 | 18:25 |
| July | 05:54 | 05:59 | 18:27 | 18:28 |
| August | 05:59 | 06:00 | 18:15 | 18:26 |
| September | 05:55 | 05:59 | 17:58 | 18:14 |
| October | 05:54 | 05:55 | 17:46 | 17:58 |
| November | 05:55 | 06:04 | 17:45 | 17:47 |
| December | 06:04 | 06:19 | 17:47 | 18:00 |
| **RANGE** | **05:48** | **06:26** | **17:45** | **18:28** |
|  |  |  |  |  |
| *www.timeanddate.com |  |  |  |  |

Table F: Coefficient values, standard errors, z-values and P-values for variables in all top spatial models.

P-values < 0.1 are italicized, with p-values < 0.05 italicized and in bold, and p-values < 0.01 italicized, bold and with an asterisk.

|  |  | **Co-efficients** | | | |
| --- | --- | --- | --- | --- | --- |
| **Top Model** | **Variables** | **Estimate** | **Std. Error** | **z value** | **P-value** |
| Human_Dog_RAI_quadratic | Human_RAI | 0.025340 | 0.277060 | 0.091 | 0.92710 |
|  | (Human_RAI)² | -0.384820 | 0.199100 | -1.933 | *0.05330* |
|  | Dog_RAI | 0.749140 | 0.386250 | 1.940 | *0.05240* |
|  | (Dog_RAI)² | -2.021460 | 0.889760 | -2.272 | ***0.02310*** |
| Distance_Settlement_quadratic | Distance_settlement | 2.553000 | 0.797700 | 3.200 | ***0.00138**** |
|  | (Distance_settlement)² | -0.000088 | 0.000031 | -2.882 | ***0.00395**** |
| Black-naped_hare_Muntjak_RAI | Black-naped hare RAI | 0.281600 | 0.128300 | 2.195 | ***0.02820*** |
|  | Muntjak RAI | 0.382100 | 0.179300 | 2.131 | ***0.03310*** |
