# Supplementary material for: Same place, different time: Leopards (Panthera pardus kotiya) employ temporal partitioning as a co-existence mechanism in a human-dominated, unprotected agricultural landscape in Sri Lanka
Source: PLoS One. 2026 Sep 9;21(9):e0335296. doi: 10.1371/journal.pone.0335296 (PMC13557350; doi:10.1371/journal.pone.0335296)
Supplement: Supplementary file 2 [file pone.0335296.s004.docx]

Response to Reviewers

We sincerely thank the Academic Editor for the opportunity to re-submit a revised manuscript for consideration to be published in PLoS ONE.

We appreciate the effort that the Editor, as well as both Reviewers, have made to help improve this manuscript and we have taken the collective advice very seriously, and addressed all comments and incorporated almost all of the suggestions (where we have not done so, we have explained our rationale).

We feel that these changes have strengthened the manuscript and that, as the first comprehensive study of its kind conducted outside protected areas in Sri Lanka, it will make an important contribution to the human-carnivore co-existence literature.

We use *italicized* text to respond to Editor and Reviewer comments below.

Thank you again,

Sincerely,

Andrew Kittle (Corresponding author)

PONE-D-25-54311

Same place, different time: Leopards employ temporal partitioning as a co-existence mechanism in a human-dominated, unprotected agricultural landscape in Sri Lanka

PLOS One

Dear Dr. Kittle,

Thank you for submitting your manuscript to PLOS ONE. After careful consideration, we feel that it has merit but does not fully meet PLOS ONE’s publication criteria as it currently stands. Therefore, we invite you to submit a revised version of the manuscript that addresses the points raised during the review process.

Thank you for submitting this fascinating manuscript. Both reviewers have many positive comments regarding the robustness and potential applications of this manuscript. However, both have also identified several minor edits required to ensure that the work is repeatable. The comments can be found in the email below.

We look forward to receiving your revised manuscript.

Kind regards,

James Edward Brereton, MSc
Academic Editor
PLOS One

Journal Requirements:

When submitting your revision, we need you to address these additional requirements.

1. Please ensure that your manuscript meets PLOS ONE's style requirements, including those for file naming. The PLOS ONE style templates can be found at

<https://journals.plos.org/plosone/s/file?id=wjVg/PLOSOne_formatting_sample_main_body.pdf> and

[https://journals.plos.org/plosone/s/file?id=ba62/PLOSOne_formatting_sample_title_authors_affiliations.pdf](https://track.editorialmanager.com/CL0/https:%2F%2Fjournals.plos.org%2Fplosone%2Fs%2Ffile%3Fid=ba62%2FPLOSOne_formatting_sample_title_authors_affiliations.pdf/1/010f019e9249fbce-bb460951-7fbf-446b-9864-e4fcaa4b852f-000000/Yy0tj-Ti-7oAVBcA6XOk3H7GvUx_QgviWYgfN4pN4bs=258)

*RESPONSE: We have gone over these forms carefully and adjusted the style of the manuscript to the requirements of PLoS ONE submissions. We couldn’t seem to find the exact instructions regarding how to submit Tables in the Supporting Documents (i.e. do these have to be separate files?) so we hope that we have done this adequately.*

2. In your Methods section, please provide additional information regarding the permits you obtained for the work. Please ensure you have included the full name of the authority that approved the field site access and, if no permits were required, a brief statement explaining why.

*RESPONSE: We have added this to the methods section under “Leopard density”*

3. When completing the data availability statement of the submission form, you indicated that you will make your data available on acceptance. We strongly recommend all authors decide on a data sharing plan before acceptance, as the process can be lengthy and hold up publication timelines. Please note that, though access restrictions are acceptable now, your entire data will need to be made freely accessible if your manuscript is accepted for publication. This policy applies to all data except where public deposition would breach compliance with the protocol approved by your research ethics board. If you are unable to adhere to our open data policy, please kindly revise your statement to explain your reasoning and we will seek the editor's input on an exemption. Please be assured that, once you have provided your new statement, the assessment of your exemption will not hold up the peer review process.

*RESPONSE: Thank you for this reminder. We have deposited the data relevant to this manuscript with the Dryad repository (DOI: 10.5061/dryad.2bvq83c5v). We have selected for it to be held privately until the peer review process is completed at which time it will be curated and made public (upon notification).*

4. We note that Figure 1 in your submission contain [map/satellite] images which may be copyrighted. All PLOS content is published under the Creative Commons Attribution License (CC BY 4.0), which means that the manuscript, images, and Supporting Information files will be freely available online, and any third party is permitted to access, download, copy, distribute, and use these materials in any way, even commercially, with proper attribution. For these reasons, we cannot publish previously copyrighted maps or satellite images created using proprietary data, such as Google software (Google Maps, Street View, and Earth). For more information, see our copyright guidelines: <http://journals.plos.org/plosone/s/licenses-and-copyright>.

*RESPONSE: Thank you for pointing this out. We have made a new Figure 1 using ArcGIS Pro and have altered the figure caption accordingly to both explain the figure and provide source information.*

5. If the reviewer comments include a recommendation to cite specific previously published works, please review and evaluate these publications to determine whether they are relevant and should be cited. There is no requirement to cite these works unless the editor has indicated otherwise.

*RESPONSE: There were no specific recommendations given by reviewers.*

6. Please review your reference list to ensure that it is complete and correct. If you have cited papers that have been retracted, please include the rationale for doing so in the manuscript text, or remove these references and replace them with relevant current references. Any changes to the reference list should be mentioned in the rebuttal letter that accompanies your revised manuscript. If you need to cite a retracted article, indicate the article’s retracted status in the References list and also include a citation and full reference for the retraction notice.

*RESPONSE: We have carefully gone through the reference list and updated the numbering after removing some references and adding others. The references that were removed were done so as a section of the discussion which focused on the perception by local communities of leopards eating dogs, was removed, as suggested by Reviewer 2. Those references were:*

*Uduman A. Towards human-leopard co-existence in Sri Lanka: social and ecological dimensions. M.Sc. Thesis, The University of British Columbia. 2020. Available from:* [*https://open.library.ubc.ca/soa/cIRcle/collections/ubctheses/24/items/1.0387338*](https://open.library.ubc.ca/soa/cIRcle/collections/ubctheses/24/items/1.0387338)*.*

*Uduman A, Hagerman S, Kroc E, Watson A, Kittle A, Burton AC. Attitudes towards the Sri Lankan leopard Panthera pardus kotiya in two rural communities. Oryx. 2022; 56(4): 528-536. doi: 10.1017/S0030605321000247.*

*Butler JRA, Linnell JDC, Morrant D, Athreya V, Lescureux N, McKeown A. Dog eat dog, cat eat dog: social-ecological dimensions of dog predation by wild carnivores. In: Gompper ME, editor. Free-ranging dogs and wildlife conservation. Oxford: Oxford University Press; 2014. Pp. 117-143.*

*Several references were also added to the manuscript in order to effectively incorporate suggestions made by both Reviewer 1 and Reviewer 2. One reference was also added to the caption of Fig. 1 which was recreated to avoid copyright infringement. These added references (in order of appearance) were:*

*World Hillshade. (2026). Esri. Retrieved from* [*https://www.arcgis.com/home/item.html?id=90dd6371b5ba4685aaf8c94abcca099e*](https://www.bing.com/ck/a?!&&p=e30d5503519e4830127d2bc450f3e91258977f40152bd57c3cd1764da360d169JmltdHM9MTc4MzQ2ODgwMA&ptn=3&ver=2&hsh=4&fclid=3c153481-6089-62d5-24e6-222d611e6359&u=a1aHR0cHM6Ly93d3cuYXJjZ2lzLmNvbS9ob21lL2l0ZW0uaHRtbD9pZD05MGRkNjM3MWI1YmE0Njg1YWFmOGM5NGFiY2NhMDk5ZQ&ntb=1)

*Otis DL, Burnham KP, White GC, Anderson DR. Statistical inference from capture data on closed animal populations. Wildife Monogr. 1978; 62: 1-135.*

*Hopcraft JGC, Anderson TM, Pérez-Vila S, Mayemba E, Olff, H. Body size and the division of niche space: food and predation differentially shape the distribution of Serengeti grazers. J. Anim. Ecol. 2012; 81: 201-213. doi: 10.1111/j.1365-2656.2011.01885.x*

*Oriol-Cotteril A, Valeix M, Frank LG, Riginos C, Macdonald DW. Landscapes of coexistence for terrestrial carnivores: the ecological consequences of being downgraded from ultimate to penultimate predator by humans. Oikos. 2015, 124: 1263-1273. doi: 10.1111/oik.02224*

*Gehr B, Hofer EJ, Muff S, Ryser A, Vimercati E, Vogt K et al. A landscape of coexistence for a large predator in a human dominated landscape. Oikos. 2017, 126:1389-1399. doi: 10.1111/oik.04182*

*Basille M, Herfindal I, Santin-Janin H, Linnell JDC, Odden J, Andersen, R et al. What shapes Eurasian lynx distribution in human dominated landscapes: selecting prey or avoiding people. Ecog. 2009, 32:683-691. doi: 10.1111/j.1600-0587.2009.05712.x*

*Klevtcova AV, Miquelle DG, Seryodkin IV, Bragina EV, Soutyrina SV, Goodrich JM. The influence of reproductive status on home range size and spatial dynamics of female Amur tigers. Mamm. Res. 2021. doi: 10.1007/s13364-020-00547-2*

*Dejeante R, Loveridge AJ, Macdonald DW, Mdhlamoto D, Valeix M, Chamaillé-Jammes. Counter-strategies to infanticide: The importance of cubs in determining lion habitat selection and social interactions. J. Anim. Ecol. 2024, 93: 159-170. doi: 10.1111/1365-2656.14045.*

*Kittle AM, Bukombe JK, Sinclair ARE, Mduma SAR, Fryxell JM. Landscape-level movement patterns by lions in western Serengeti: comparing the influence of inter-specific competitors, habitat attributes and prey availability. Move. Ecol. 2016, 4:17. doi: 10.1186/s40462-016-0082-9*

Reviewers' comments:

**Comments to the Author**

Reviewer #1: This study provides insights into leopard density, site use, and activity patterns within the human-dominated Central Highlands of Sri Lanka, where tea estates dominate the region. The study fills a critical knowledge gap in the ecology of carnivores and their coexistence outside of protected areas and is highly relevant to the field of carnivore research. Overall, the study is technically sound, and the data adequately support the study’s interpretations and conclusions.

*RESPONSE: Thank you for your positive comments and interest in the manuscript.*

However, as the protected-area datasets were collected during different time periods than the tea estate dataset, the author should also highlight the potential impact of seasonal or temporal variations on the activity patterns, as it might partially affect the differences observed.

*RESPONSE: Thank you for this comment as seasonal patterns may be important for leopard activity times. However, all three of the datasets do in fact overlap in terms of time of year with the tea estate study from August through November 2016, the Wilpattu NP study from July through October 2015 and the Gal Oya NP study including data from > 2 years (November 2017 – June 2020). Nevertheless, we have explicitly addressed the imperfect overlap within the text in the discussion section under Leopard activity times: “It is important to note that there was not perfect overlap regarding the time of year monitored for all 3 sites; that GONP was monitored for considerably longer than both WNP and the tea estate landscape (20 months vs 4 months); and that the sites are all located within different climatic zones. As such, there may be additional factors which partially underlie these observations. However, the strong similarities in activity patterns detected between WNP and GONP, despite having varied monitoring periods and being in different climatic zones, supports the conclusion that leopards in the tea estate landscapes are indeed acting differently and employing temporal partitioning to avoid people.”*

Reviewer #2:

Title and Abstract:

The title would benefit from capitalising major words for consistency with scientific style and including the scientific name at first mention.

*RESPONSE: We actually did this, and then looked at the style guide for PLoS ONE and realized it calls for “sentence style” with only the first word capitalized. So we have left it as it was, except for adding the scientific name.*

The abstract contains several long sentences that reduce readability; tightening these and presenting the key findings more concisely would improve accessibility.

*RESPONSE: Thank-you for this suggestion. We have attempted to improve readability in several ways. We have broken up the first sentence into 2 parts (Ln 14 -16). In Ln 16 we add a comma to more effectively break up the sentence. In Ln 26 we change “human-dominated” to “shared” to improve flow. In Ln 29 we break the sentence into two parts to improve flow. In Ln 31 we remove a redundant comment “and their importance” to make the sentence more concise.*

The scientific name should also appear upon first mention in the abstract.

*RESPONSE: Ok, this has been done.*

Introduction:

The discussion of global protected areas (lines 37–48) feels broader than necessary for a study centred on Sri Lanka. This section could be more concise and more directly linked to the local context.

*RESPONSE: Thank you for this suggestion. We have substantially reduced this paragraph (174 to 151 words) by removing specific references to individual taxa and instead speaking more broadly about the importance of unprotected areas for wildlife. We have also removed some unnecessary wording (“It is widely accepted that…”).*

*With regards to linking explicitly to the local context, we don’t feel that this is the right place to do it as what we intended here was to make the argument that broadly, how wildlife utilize unprotected regions need to be better understood in general, given increasing human impacts and climate change-induced movements. The link to the local context comes later. We hope this rationale is satisfactory.*

The section on human–wildlife conflict (lines 49–66) is relevant but currently repetitive; streamlining it would improve flow.

*RESPONSE: Thank you for this suggestion. We have reduced this section as well (from 262 words to 225) by drastically compressing the opening sentence and removing redundant and unnecessary wording (e.g. replace “these large predators” with “they”; remove “it is increasingly accepted that…”; remove “in terms of”, “furthermore”; remove duplicate statements about explicitly sharing space” etc).*

The term “top carnivore” reads awkwardly and could be replaced with “apex predator” for clarity and consistency with later usage.

*RESPONSE: Thank you for this suggestion. It makes much more sense to use “apex predator” and we have done this.*

Lines 67–78 could also be condensed.

*RESPONSE: Thank you for this suggestion. We have likewise reduced this section from 171 words to 153, by removing redundancies and being more concise.*

The material in lines 79–93 provides the clearest initial framing of the study and may be more effective if moved earlier.

*RESPONSE: While we see the reviewer’s point in terms of its clarity for the purposes of this study, we have attempted to frame the research by narrowing from the broad scale to the specific, and feel that this section, which deals with the specific study area under investigation should come at the end of the Introduction. We hope that this is acceptable.*

“UNESCO” should be written in full on first use (line 84).

*RESPONSE: Ok, this has been done.*

Finally, the use of first person (“we”) in lines 94–103 should be avoided to maintain an objective tone.

*RESPONSE: Ok, this has been done.*

Materials and Methods:

The Methods are generally strong, detailed, and scientifically rigorous, though several areas would benefit from clarification.

*RESPONSE: Thank you for this positive assessment of the Methods section. We have attempted to improve the clarity of the section as suggested.*

The study area description (lines 106–131) contains extensive climatic detail, but the relevance of specific rainfall patterns should be explained more clearly in relation to leopard ecology or study design.

*RESPONSE: We thank the reviewer for this pertinent point. On reflection, we realize that the detailed climatic information is not highly relevant to this study – or to leopard ecology in general given their ability to thrive across a wide range of climates. Our intention had been to describe the study site to situate the reader, but realize that we went overboard on detail. As such, we have condensed this section (from 189 words to 161) to provide an overview, hopefully without burdening the reader with too much unnecessary detail.*

The explanation of estate structure and settlement patterns (lines 128–149) is useful but would be strengthened by explicitly linking these features to expected leopard behaviour.

*RESPONSE: We thank the reviewer for this insightful comment also, which again forced us to reflect on our description. The key element we are trying to get across is that this landscape has an unusually broad human footprint with people spread widely across the landscape. The impact of this is that wildlife cannot effectively separate spatially from humans at a broad scale as it can in areas where human populations are high but clustered. We have therefore added to the sentence claiming that “the human footprint is large and extensive” to clarify this: “As such, the human footprint in this landscape is large and extensive, making broad-scale spatial separation between humans and wildlife impossible.”*

The camera trap design (lines 150–173) is well justified, though the rotation of cameras across three rounds (lines 138–144) requires clearer explanation to ensure the population closure assumption is met.

*RESPONSE: Thank-you for the positive feedback. Regarding the rotation of cameras, we have added a sentence explicitly detailing the duration of the entire study (all 3 rounds combined) and justifying (with references which link it to a study which provides a biological underpinning) this duration as fitting within a closed population. In addition, we have included specific mention of running the ‘closure’ test in ‘secr’.*

The decision to use only left flank images (lines 159–166) is reasonable, but the potential impact of excluding individuals should be acknowledged.

*RESPONSE: Ok, thank you. We have added a couple of explanatory sentences as follows: “Employing only a single flank has the potential to result in individuals being excluded which can impact density estimates. However, the fact that the three images excluded were lacking in clarify sufficient to unambiguously identify or sex the individuals, alleviates this concern.”*

The SECR modelling approach (lines 167–196) is robust, though the effort weighting method (line 175) is unconventional and would benefit from justification or citation.

*RESPONSE: Ok, we have added a citation for an earlier publication which utilizes this same weighting method (Kittle AM,* *Watson AC, Samaranayake PKL. Edge effects and distribution of prey forage resources influence how an apex predator utilizes Sri Lanka’s largest protected area.* *J. Zool. 2021; 314(1): 31 – 42. doi: 10.1111/jzo.12870) and added it to the existing citation (Efford MG, Borchers DL, Mowat G. Varying effort in capture-recapture studies. Methods Ecol. Evol. 2013; 4(7): 629-636. doi: 10.1111/2041-210X.12049) for the theoretical underpinning.*

The iterative buffer selection process (lines 174–196) is thorough but could be summarised more concisely, with full details placed in supplementary material.

*RESPONSE: Ok, thank you for this suggestion. We have summarized this section in the main text by alluding only to the general process employed and directing readers to Table S2 for details. We have then moved the more detailed description to the caption for Table S2 (which shows the iterative buffer size estimation process). This has streamlined this section considerably.*

The site utilisation analysis (lines 197–243) is clearly structured, though the use of Google Earth for settlement distance (lines 200–205) should be justified

*RESPONSE: Thank you for the comment. We attempted to justify this method in the original ms by stating that “available GIS layers did not have sufficiently fine grain to identify all communities on the landscape”. However, we have more clearly justified this in the revision by stating: “Distance to human settlement was determined using the “distance measure” tool on Google Earth because many estate communities are small clusters of dwellings which are not represented on available GIS layers, but are visible on Google Earth maps.”*

, and the treatment of plantation forests as equivalent to natural forest (lines 214–220) may require ecological explanation.

*RESPONSE: Thank you for this comment. As above, we attempted to explain this in the original manuscript as follows: “forests were considered all treed landscapes inclusive of plantation forests as leopards are generalists [58] and these habitats are used by leopards, especially in landscape mosaics [59]” with the citations supporting the justification. However, we realize that this was insufficient and poorly worded. We have now tried to further clarify this approach by stating:* *“forests were considered all treed landscapes inclusive of plantation forests, which here are typically Eucalyptus-dominated stands originally planted to provide fuelwood for the tea factories. Natural and plantation stands were treated as the same since leopards are adept at utilizing a wide range of habitats [58] including plantation patches, which are especially important in landscape mosaics [59] despite their inferior quality from a biodiversity perspective”*

The handling of multicollinearity (lines 221–243) is appropriate, though the rationale for selecting quadratic terms (lines 221–223) could be strengthened.

*RESPONSE: Thank you for this comment. We agree that this wording was a little weak. We have added two references here to strengthen the support for this approach (Balme GA, Hunter LTB, Slotow R. Feeding habitat selection by hunting leopards Panthera pardus in a woodland savanna: prey catchability versus abundance. Anim. Behav. 2007; 74(3): 589-598. doi: 10.1016/j.anbehav.2006.12.014 and Hopcraft JGC, Anderson TM, Pérez-Vila S, Mayemba E, Olff, H. Body size and the division of niche space: food and predation differentially shape the distribution of Serengeti grazers. J. Anim. Ecol. 2012; 81: 201-213. doi: 10.1111/j.1365-2656.2011.01885.x). We also added an explanatory sentence: “If continuous variables are acting quadratically, model coefficients for the quadratic term should be negative, which indicates that although a leopard might select for a particular feature (e.g. elevation) or resource (e.g. dog abundance), it avoids areas where the feature is extreme or resource overabundant, resulting in a non-linear relationship [63].”*

The activity time analysis (lines 244–287) is well explained, but the Z test comparisons (lines 267–287) may require mention of multiple comparison considerations.

*RESPONSE: Thank you for this comment. We have added some explanatory detail here to make clear that there was more than one comparison undertaken, so that the description of the method now reads: “To compare leopard activity patterns between the protected (WNP and GONP) and unprotected (tea estate landscape) areas, we compared the independent proportions of leopard observations for each of the time periods between the unprotected site and each protected site in turn.”*

Overall, the Methods are sound and replicable, and clarifying a few analytical decisions would further strengthen the study.

*RESPONSE: Thank you for this positive and supportive comment. We have tried to address the reviewers concerns and improved clarification of the analytical decisions that were pointed out. We agree that this improves the interpretability of the manuscript.*

Results:

The Results are clearly organised and generally well presented.

*RESPONSE: We appreciate that the reviewer felt this was the case.*

The density results (lines 288–304) are straightforward, with the top model clearly identified and the buffer justified, though the large difference between the observed and model estimated sex ratios (lines 293–299) should be addressed in the Discussion.

*RESPONSE: Thank you for bringing up this important point. We have addressed the large observed difference between the observed and model estimates sex ratios more directly than we had previously in the Discussion section about adult sex ratios as follows: “This model allowed for variation between sexes to influence the spatial parameter related to home range size (σ), but not the probability of capture at home range centre (g0). The much larger σ seen for males compared to females (x̅ = 2409 vs 737) indicates substantial home range size variation resulting in an even more female-biased ASR than was apparent from raw observations, as the latter would have likely included observations of males far from home range centres.”*

The comparison with other Sri Lankan studies (lines 301–316) is useful, but the text could more directly state that overlapping confidence intervals indicate no significant difference in density.

*RESPONSE: Although we felt that we had been quite clear about this in the original ms, we have altered the language slightly to make the implication even more clear. It now reads: “The observed density was lower than what has been found in Sri Lanka’s best protected National Parks, but in almost every case 95% CIs overlap, indicating that this difference is not significant (Table 3)”. We hope that this small change is sufficient to clarify the issue.*

The site use findings (lines 317–324) are well linked to the hypotheses, showing that overall leopard use was highest at intermediate human and dog abundance, males preferred intermediate distances from settlements, and females selected areas with higher prey availability. These patterns are well explained, though the relatively low R² values should be acknowledged.

*RESPONSE: Thank you for this positive assessment. We are glad that the site use findings are clear. We have addressed the low R2 values in the discussion at the end of the “Leopard site utilization” section, where we indicate that this suggests that influential variables (e.g. social, climatic factors etc.) might not have been included in the models and urge follow up research to allow for a more holistic understanding of the factors driving leopard space use in this system. Specifically, we wrote: “It is important to recognize that the R² values of the top site utilization models were modest (0.16 – 0.26) which suggests that important influencing variables were not included in the modelling procedure. Ecological systems are complex with numerous environmental, anthropogenic, physiological and social factors that influence how predators utilize their habitat and even key factors can be overlooked or unmeasured. For example, social interactions between leopards such as mating and cub-rearing can alter space use decisions in both the short- and longer term [96,97], while seasonal climatic factors can impact site use on a cyclical basis [98]. As such, it is important to build on studies such as this one in an attempt to reach a more holistic understanding of leopard space use decisions and their underlying factors.”*

Discussion:

The Discussion is thoughtful and well connected to the results, though several sections could be tightened for clarity and impact.

*RESPONSE: Thank you for this positive assessment. We have attempted to address all of the areas which needed to be improved.*

The opening discussion of density (lines 352–367) effectively compares findings with protected areas, but would benefit from explicitly noting that overlapping confidence intervals do not necessarily imply ecological equivalence.

*RESPONSE: Thank you for this important comment. We have taken your advice and explicitly stated that these overlapping 95% Cis do not imply ecological equivalence between PAs and unprotected landscapes, but do suggest that some unprotected landscapes may also be suitable habitats for leopards.*

The comparison with recent high density estimates (lines 368–392) is important, though the critique of methodology could be more concise, and the suggestion of overcrowding in protected areas may require stronger evidence.

*RESPONSE: Ok, we have made the methodology critique more concise (84 to 72 words). We have also altered the section about what these high densities, if correct, might mean, including suggesting increased populations within PAs due to outside pressures as one option. In fact, we have removed the reference to overcrowding –since, as the reviewer suggests, this is very difficult to accurately determine - and focused on the possibility that PAs become increasingly important refuges when the surrounding landscape becomes increasingly degraded.*

The explanation of the female biased sex ratio (lines 392–416) is well supported but could be summarised more succinctly.

*RESPONSE: Thank you for this positive feedback. We have tried to make this section more concise (word count from 260 to 223).*

The site utilisation discussion (lines 417–439) interprets the quadratic relationships with human and dog presence clearly, though the section could more directly connect these findings to coexistence mechanisms rather than focusing heavily on community perceptions of dog predation.

*RESPONSE: Thank you for this useful comment. We agree that it is more effective to connect these observations to coexistence mechanisms, and we have attempted to do that by linking results directly to the landscape of co-existence concept (Ln 453 to 456). We have also introduced some supporting literature to strengthen these ideas. We decided also to separate the human use and domestic dog use results to increase clarity and have significantly reduced the discussion surrounding community perceptions of dog predation.*

The distinction between male avoidance of settlements and female prey driven space use (lines 417–436) is well articulated but could be made more concise.

*RESPONSE: Ok, we have made this more concise and shortened the word count from 201 to 165.*

The activity time interpretation (lines 440–462) convincingly demonstrates temporal partitioning, though the explanation of reduced diurnal activity could be streamlined, and the comparison with Nepal may be more effective if shortened.

*RESPONSE: Thank you for this comment. We have considerably shortened this section (184 to 125 words) making it much more concise and, we hope, effective.*

The final section on snares (lines 462–end) is strong and policy relevant, and would benefit from a clearer link back to the central argument that behavioural adaptations alone cannot offset human caused mortality.

*RESPONSE: Thank you for this valuable point. We have added a section which reads “…and provide a stark reminder that behavioural adaptations by wildlife are not, on their own, sufficient to offset human-induced mortality”.*

Overall, the Discussion is robust and insightful, and with some tightening it will read as a confident, cohesive, and persuasive interpretation of your findings.

*RESPONSE: Thank you for this positive assessment. We have endeavored to tighten up the discussion as much as possible to make it more streamlined and coherent.*
